# Degradation of the Indospicine Toxin from *Indigofera spicata* by a Mixed Population of Rumen Bacteria

**DOI:** 10.3390/toxins13060389

**Published:** 2021-05-28

**Authors:** Rosalind A. Gilbert, Gabriele Netzel, Kerri Chandra, Diane Ouwerkerk, Mary T. Fletcher

**Affiliations:** 1 Department of Agriculture and Fisheries, EcoSciences Precinct, Dutton Park, QLD 4102, Australia; kerri.chandra@daf.qld.gov.au (K.C.); Diane.Ouwerkerk@daf.qld.gov.au (D.O.); 2 Queensland Alliance for Agriculture and Food Innovation, The University of Queensland, St Lucia, QLD 4072, Australia; g.netzel@uq.edu.au (G.N.); mary.fletcher@uq.edu.au (M.T.F.)

**Keywords:** indospicine, *Indigofera*, detoxification, rumen, bacteria, fermentation

## Abstract

The leguminous plant species, *Indigofera linnaei* and *Indigofera spicata* are distributed throughout the rangeland regions of Australia and the compound indospicine (L-2-amino-6-amidinohexanoic acid) found in these palatable forage plants acts as a hepatotoxin and can accumulate in the meat of ruminant livestock and wild camels. In this study, bovine rumen fluid was cultivated in an in vitro fermentation system provided with *Indigofera spicata* plant material and the ability of the resulting mixed microbial populations to degrade indospicine was determined using UPLC–MS/MS over a 14 day time period. The microbial populations of the fermentation system were determined using 16S rRNA gene amplicon sequencing and showed distinct, time-related changes occurring as the rumen-derived microbes adapted to the fermentation conditions and the nutritional substrates provided by the *Indigofera* plant material. Within eight days of commencement, indospicine was completely degraded by the microbes cultivated within the fermenter, forming the degradation products 2-aminopimelamic acid and 2-aminopimelic acid within a 24 h time period. The in vitro fermentation approach enabled the development of a specifically adapted, mixed microbial population which has the potential to be used as a rumen drench for reducing the toxic side-effects and toxin accumulation associated with ingestion of *Indigofera* plant material by grazing ruminant livestock.

## 1. Introduction

The *Indigofera* plant species are deep-rooted, perennial shrubs that are highly palatable to ruminant livestock and are considered endemic in many countries, particularly those in subtropical and tropical regions [1,2]. Although *Indigofera* are widely distributed, some *Indigofera* species (e.g., *Indigofera spicata*) were introduced into mainland USA and Australia as high-protein pasture legumes due to their ability to tolerate drought, floods and salinity, before their potential toxicity was recognised [3,4]. Of the many *Indigofera* species now present in Australia, at least eight have been found to contain the non-acute hepatotoxin indospicine [5,6], which was demonstrated in rodent studies to be the responsible toxin by subcutaneous injection of the isolated amino acid [7]. Indospicine is an arginine analogue, which, rather than being incorporated into proteins, persists as a free amino acid which can accumulate in tissues of animals who have ingested *Indigofera* plant material [8,9,10]. Several species of herbivores have been shown to bioaccumulate indospicine including horses, goats, cattle, rabbits and camels, resulting in poor animal performance, reduced weight gain, reproductive losses and mild to severe liver disease [1]. In addition, indospicine represents a potential food safety issue, with domesticated dogs suffering liver damage following ingestion of indospicine-contaminated meat [11,12,13,14].

**Figure 1:**
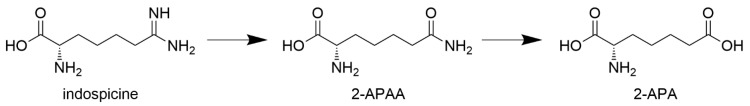
Chemical structures of indospicine and metabolites, 2-aminopimelamic acid (2-APAA) and 2-aminopimelic acid (2-APA).

While previous studies have suggested that indospicine contamination of meat can be managed by thermo-alkaline treatment [15], an alternative management approach may be to increase the rate at which the indospicine contained in plant fodder can be broken down in the animal gut (rumen) and thus reduce the concentrations of indospicine which accumulate in the muscle tissue. Previous in vitro studies have shown that indospicine can be broken down by microbes sourced from the foregut of camels and rumen of cattle to form the breakdown products, 2-aminopimelamic acid (2-APAA) and 2-aminopimelic acid (2-APA) (Figure 1) [16]. The study indicated that the rate of this degradation may be relatively slow, requiring up to 48 h incubation, and therefore the rate at which these microbes utilise indospicine may not be considered fast enough to prevent the water-soluble indospicine from moving from the forestomach or rumen into the intestine, where it can be absorbed and accumulated in the tissues [16,17]. The metabolites 2-APAA and 2-APA are amino acids and do not demonstrate the same propensity to accumulate. Although low levels of 2-APAA and 2-APA have been detected in animals fed *Indigofera*, these levels did not persist after cessation of *Indigofera* consumption [17].

Fermentations conducted in vitro and seeded with rumen fluid sourced from animals have often been conducted in order to cultivate difficult-to-grow organisms and experimentally ascertain the effects of alternative feeds, plant extracts and specific compounds on rumen microbial populations [18,19,20,21,22]. These mixed microbial fermentations may be undertaken using either (1) relatively small-scale, short-term batch cultures which provide good experimental replication, or (2) single- or multi-chamber chemostats to better simulate rumen-like conditions and enable longer-term measurement of fermentation parameters, for example, the Rumen Simulation Technique (RUSITEC) apparatus [22,23,24,25]. Using a mid-sized single-chamber continuous anaerobic fermentation system, rumen-derived microbial communities have been successfully cultivated in order to reduce the toxicity of the ruminant fodder crop *Leucaena leucocephala* [26,27]. *Leucaena* is a high-protein tropical leguminous shrub which contains the toxic amino acid, mimosine. While mimosine is readily broken down by many genera of rumen bacteria, the intermediates in the metabolism of mimosine, dihydroxypyridines (DHP), are also toxic and are degraded only by the slower-growing, less abundant rumen bacteria classified within the phylum *Synergistetes*, specifically the species *Synergistes jonesii* [28,29]. While early studies involved the transfer of rumen fluid between animals in order to establish microbial populations able to detoxify DHP [26,27], the mixed microbial population prepared using a single-chamber in vitro fermentation system has been supplied as an intra-ruminal drench to prevent toxicity effects developing in cattle maintained on pasture and *Leucaena* forage [30,31].

This study aimed to examine whether microbial populations capable of detoxifying indospicine could be enriched and cultivated in a similar in vitro single-chamber anaerobic fermentation system. This study also sought to develop new methodology required to formulate a mixed microbial inoculum for reducing the toxic side-effects and accumulation of indospicine in domesticated herbivores grazing pastures containing *Indigofera*.

## 2. Results

### 2.1. Fermenter: Concentrations of Indospicine and Breakdown Products

**Figure 2:**
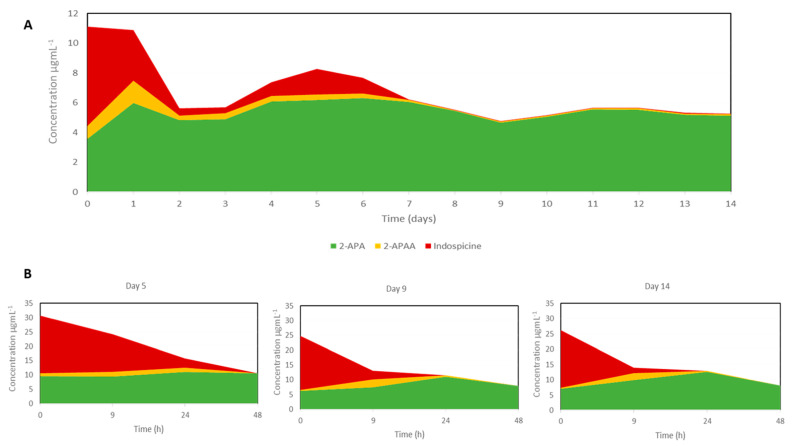
Degradation of indospicine during the fermentation trial. (**A**) Concentrations of indospicine, 2–APAA and 2–APA in daily subsamples collected over the 14 days of the in vitro fermentation; and (**B**) degradation assays of fermenter fluid subsampled on fermentation days 5, 9 and 14. Indospicine (*Indigofera* extract) was added to fermenter fluid and incubated at 39 °C and sub-samples analysed after 0, 9 and 24 and 48 h of incubation.

*Indigofera spicata* plant material utilised in this study showed a high-protein nutritional analysis (Table 1) as expected of a legume and an indospicine content of 1310 mg kg^−1^. Indospicine was continually supplied to the fermenter via the daily addition of 10 g dried *I. spicata* plant material. Replicate analyses of fermenter fluids collected daily immediately prior to the addition of dried *I. spicata* plant material were carried out by ultra-performance liquid chromatography–tandem mass (UPLC–MS/MS) with an LOQ of 0.02 µg mL^−1^ for each of indospicine (CV ≤ 6%), 2-APAA (CV ≤ 10%) and 2-APA (CV ≤ 10%). Although indospicine was continually supplied to the in vitro fermenter, the concentration of indospicine within the fermenter was shown to decline during the first seven days of the fermentation. Indospicine degradation reached 100% by the eighth day of fermentation (Figure 2, Supplementary Material Table S1), as indicated by the UPLC–MS/MS analysis of fluid collected from the fermenter on a 24 h basis, immediately prior to daily addition or “feeding” of ground *I. spicata* plant material. Of the end-products of indospicine degradation, 2-APA remained at a relatively constant concentration throughout the 14 days of fermentation. In contrast, although the intermediate degradation product 2-APAA was consistently detected at low concentrations, after eight days of fermentation, 2-APAA concentrations further declined and remained at very low concentrations (≤0.10 µg mL^−1^ or ≤0.32 mg per 3 L total fermentation volume).

A concentrated aqueous extract of *I. spicata* (146.3 mgL^−1^ indospicine) was prepared and used in our indospicine degradation assay. Samples of fermenter fluid were collected throughout the fermentation (day 0, 5, 9 and 14) and tested for their ability to degrade indospicine in in vitro degradation assays, showing that the microbial populations cultivated in the fermenter were able to degrade indospicine within 24 h of incubation at 39 °C (Figure 2B). These degradation assays were carried out in triplicate with an LOQ of 0.05 ug mL^−1^ for each of indospicine (CV ≤ 24%), 2-APAA (CV ≤ 16%) and 2-APA (CV ≤ 20%). The rate at which the fermenter fluid could degrade indospicine, however, increased with time of fermentation. The initial fermenter fluid sample collected on day 5 had a higher concentration of indospicine remaining after 9 h of incubation than the fermenter fluid samples collected on days 9 and 14.

### 2.2. Fermenter: Microbial Community Diversity

The microbial community diversity occurring within the sample (alpha diversity) of the cryopreserved bovine rumen fluid used to inoculate the fermenter systems with microbes, was far higher than the microbial diversity determined for the end of the 14 day fermentation period. The mean diversity of duplicate, technical replicates of the rumen fluid sample were 963, 9.18 and 61.42 for the observed species, Shannon and Faith phylogenetic diversity (PD) measures. In contrast, for duplicate samples of the fermenter fluid collected on day 14, the mean diversity for each these measures decreased to 563.5, 7.85 and 46.39, respectively.

**Figure 3:**
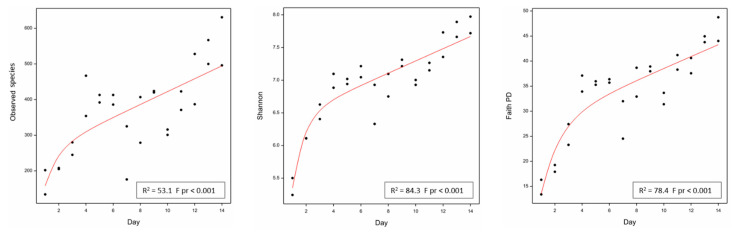
Significant changes in microbial diversity with time of fermentation as indicated by three diversity measures (Observed species, Shannon and Faith phylogenetic diversity (PD) indices). Diversity measures for two replicate samples collected on each day of the fermentation are plotted (**●**) and a line fitted using a line plus exponential regression model (R^2^ and F pr values indicated).

Statistical analyses of microbial diversity indices (observed species, Shannon and Faith phylogenetic diversity (PD)) showed significant changes in microbial diversity occurring with increased time of fermentation (F pr < 0.001 for each measure). This effect was most obvious for the measures of Shannon and Faith PD. For example, for the Shannon index, diversity was shown to initially increase exponentially from approximately 4 to 6 days of fermentation, and then increasing linearly beyond that (Figure 3; Supplementary Material Table S2). While this linear increase would be unlikely to continue indefinitely given the closed environment of the fermentation apparatus, extending the fermentation time beyond 14 days may have resulted in further increases in microbial community diversity.

### 2.3. Fermenter: Microbial Populations

**Figure 4:**
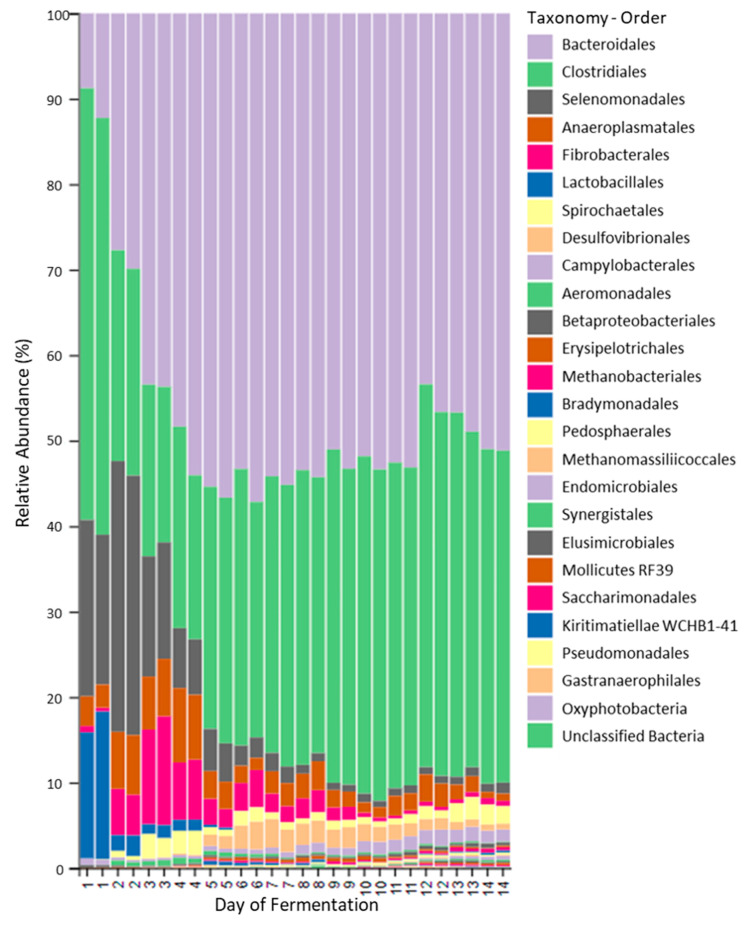
Time-related changes occurring in the bacterial and archaeal populations present in the *I*. *spicata* fermentation. Highly abundant bacterial and archaeal populations (sequence numbers > 3rd percentile), identified in replicate fermenter fluid samples collected on each day of the fermentation, classified according to order level.

The microbial community of the fermenter changed considerably with time, with the most changes occurring within the first five days of the fermentation (Figure 4). When the bacterial and archaeal populations of the original rumen fluid sample used to inoculate the fermentation were compared with the those present in the fermenter fluid collected on the final day of the fermentation (day 14) using a Venn graph approach, approximately 37% of identified microbial species (Feature) present in the original rumen fluid sample, were no longer detected. Almost half (47.3%) of the microbial species present in the original rumen fluid sample, however, were still detected in fermenter fluid after 14 days of fermentation. In addition, the fermenter fluid collected on day 14 contained 29 unique species (15.9% of total populations). These unique species are presumably either microbial populations present at very low, undetectable concentrations in the original rumen fluid sample, or microbial populations introduced into the fermentation during the course of the fermentation. As the fermentation vessel was maintained in a relatively aseptic manner, these additional populations may have originated from the plant material provided. The most highly abundant (>100 sequences per Feature) unique species identified at the end of the fermentation, included those classified in the genera *Lachnoclostridium*, *Endomicrobium* and *Tyzzerella* 3 and the families *Ruminococcaceae* and *Dysgonomonadaceae*. Interestingly, the genus of secondary fermenters, *Pyramidobacter* (phylum Synergistetes) which was not detected in the original rumen fluid sample, increased in abundance within the fermenter from day 4 onwards (77 sequences detected in fermenter fluid from day 14).

**Figure 5:**
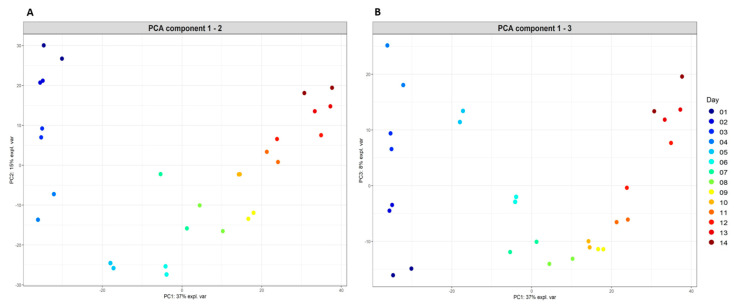
Changes in microbial populations of the *Indigofera* fermentation occurring within a 14 day time period: principal component analysis (PCA) of bacterial and archaeal populations; (**A**) PCA component 1–2; (**B**) PCA component 1–3.

Of the microbial populations maintained in the fermentation, the most highly abundant phyla included Bacteroidetes and Firmicutes, with populations classified within the order Bacteroidales (uncultured Bacteroidales F082), Rikenellaceae RC9 gut group, genus *Prevotella* and family Lachnospiraceae (e.g., genus *Butyrivibrio*) present in all fermenter fluid samples analysed and dominating by the 14th day of the fermentation. When the total microbial populations present in replicate fermenter fluid samples collected daily were plotted spatially according to the extent of microbial community similarity (Figure 5), distinct time-related changes in microbial community structure were observed. This analysis further indicated that the microbial populations initially present in the rumen fluid sample used to inoculate the fermenter, changed in response to time spent in the fermenter, with some microbial taxonomic groups increasing in relative abundance while other microbial populations declined. The most highly abundant, core taxonomic groups observed during the later stages of the fermentation, when the microbial populations were most stable (from day 8 of the fermentation), were predominated by genera classified within the orders Bacteroidales and Clostridiales (Table 2).

**Figure 6:**
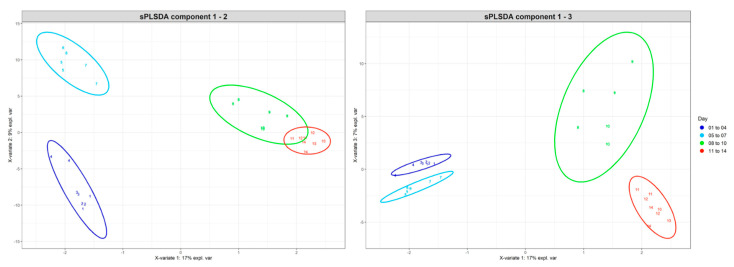
Sparse Partial Least Squares Discriminant Analysis (sPLSDA) of microbial populations of fermenter fluid samples collected over a 14 day time period, with samples collected daily grouped together and coloured (days 1–4, 5–7, 8–10 and 11–14) and 95% confidence level ellipses shown for two sPLSDA component plots (components 1–2 and 1–3).

The microbial populations or distinct taxonomic groups contributing to the time-related differences observed were determined using Sparse Partial Least Squares Discriminant Analysis (sPLSDA) with data for the duration of the fermentation subdivided into four time periods (days 1–4; 5–7; 8–10 and 11 to 14) (Figure 6; Supplementary Material Table S3). Methanogenic archaea were not found to be highly abundant throughout the fermentation and did not contribute to any of the time-related differences observed using sPLSDA. Of the two families of archaea detected in the fermenter (Methanobacteriaceae and Methanomethylophilaceae), the populations of *Methanobrevibacter* were shown to be the most highly abundant archaeal genus present after 14 days of in vitro fermentation (158 sequences detected in fermentation fluid from day 14).

**Figure 7:**
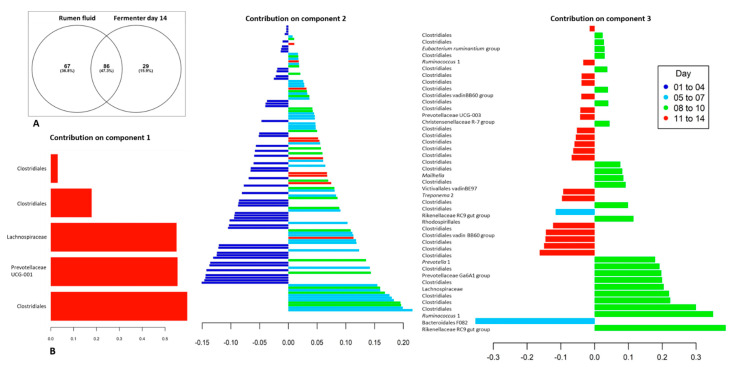
Time-related differences in bacterial and archaeal communities: (**A**). Venn diagram of shared and unique populations (Features) identified in the rumen fluid used to inoculate the fermentation (Rumen fluid) and the last day of the fermentation (day 14); and (**B**). Sparse Partial Least Squares Discriminant Analysis (sPLSDA) showing the microbial populations (OTU Features) contributing to the differences occurring between grouped days (days 1–4, 5–7, 8–10 and 11–14). The positive or negative contribution of each microbial population to the first, second and third component are indicated on the x axis, with the relative contributions of each microbial group ranked from bottom (important) to top. Colours indicate the time period (days) of the fermentation, in which the microbial populations were most abundant. Microbial populations contributing to the variance of each component, including component 2, are listed in full taxonomic detail in Supplementary Table S1.

Bacterial populations which significantly contributed to the differences occurring between the earlier days (days 1 to 4) (Figure 7B sPLSDA component 2 plot) and later days of the fermentation included many populations classified within the phylum Firmicutes, order Clostridiales, with the majority of these populations not classified further than the taxonomic level of order. Additional orders contributing to these differences included Selenomonadales (*Anaerovibrio*), Lactobacillaceae (*Lactobacillus mucosae*) and Bacteroidales (Prevotellaceae YAB2003 group). Similarly, bacterial populations significantly contributing to the differences occurring between days 5 and 7 and other days were mainly classified within the order Clostridiales (including representatives of the families Ruminococcacaea and Lachnospiracaea) and the phyla Spirochaetes (*Treponema*) and Bacteroidetes. Populations present at days 8 to 10 differed from the other days, not only in the populations of Clostridiales present (for example, *Ruminococcus*), but in populations of Bacteroidetes classified within the Rikenellaceae RC9 gut group and the Prevotellaceae Ga6A1 group. By the last four days of the fermentation (days 11 to 14), populations of Clostridiales were still contributing to the observed differences in fermenter populations (Figure 7), as were populations of Prevotellaceae, the Rikenellaceae RC9 gut group and the phylum Spirochaetes (*Treponema*).

## 3. Discussion

Results of the chemical analysis of indospicine levels by UPLC–MS/MS, showed that the microbial populations cultivated within the in vitro anaerobic fermentation system could effectively breakdown the indospicine toxin, continuously provided in the form of *Indigofera* plant material. The rate at which the microbial populations of the fermenter degraded indospicine increased with time; and within eight days of fermentation, all the indospicine provided was being degraded within a 24 h period. While no mammalian enzymes have been reported to be able to degrade the unusual amidino group present in indospicine, which instead inhibits nitric oxide-mediated enzymatic functions [10,32], the ability of microbes present within the herbivore gut to degrade and metabolise indospicine and form the amine and acid breakdown products (2-APAA and 2-APA) has been previously shown experimentally [16]. This former study established that indospicine could be degraded by microbial populations sourced from the forestomach of camels and bovine rumen fluid within 48 h of incubation in vitro. A further time-dependent in vitro degradation experiment conducted using camel forestomach fluid only, showed that indospicine degradation occurred most rapidly during the first 8–18 h of incubation (65 nmolh^−1^) and it was suggested that bovine rumen microflora may have the capacity to degrade indospicine at a similar rate [16]. It is worth noting that the metabolites 2-APAA and 2-APA are one-carbon homologs of the more common amino acids glutamine and glutamate, and the metabolism of 2-APAA to 2-APA through loss of NH_3_ from the amide group is thus analogous to the same metabolism of glutamine to glutamate. Glutamate is readily further metabolised to 2-ketoglutarate and other metabolites with oxidation to carbon dioxide being a major metabolic fate. It would, therefore, be assumed that 2-APA is similarly oxidized and metabolised to a range of smaller metabolites including carbon dioxide.

In the current investigation, where a bovine rumen fluid sample was used as the sole source of microbes for a continuous fermentation system, the ability of microbial populations to degrade indospicine was determined by in vitro assays conducted in a time-dependent manner. Following five days of fermentation, fermenter fluid was shown to degrade indospicine at a rate similar to that observed in experiments with camel foregut fluid (99.4% indospicine degraded after 48 h of incubation), with only 35% of the indospicine degraded in the first nine hours. In contrast to the camel-based study, however, degradation assays using fluid subsampled on subsequent days of the fermentation (days 9 and 14) showed a large increase in the rate at which indospicine was degraded. By the 14th and final day of the fermentation, 90% of the indospicine was degraded within nine hours of incubation, with the remaining 10% completely degraded within 24 h. These findings, therefore, demonstrated that not only could the fermentation system successfully maintain the growth of microbial populations able to degrade indospicine, the system promoted the growth of microbial populations with the functional capacity to more rapidly degrade indospicine. The observed 100% degradation of indospicine within 24 h (Figure 2B) compares favorably with mean retention time of ingesta in the rumen of ruminant animals eating a range of diets of approximately 25 h as reported by other researchers [33,34], thus supporting the proposal that a rumen drench could be developed for practical application to ruminant livestock.

Sequence-based microbiome analysis of the microbial populations proliferating in the fermenter, indicated that bacterial and archaeal populations changed with time, as they responded to the physical and nutritional conditions provided by the in vitro fermentation system. The bovine rumen fluid used to provide the initial microbial communities of the fermenter was obtained from a steer maintained on improved pasture with hay supplementation. It has been well established that within an animal, diet is the primary driver of rumen microbial communities [35,36]. The provision of an alternative diet to that supplied to the animal from which the initial rumen fluid was obtained, may have contributed to the distinct changes in microbial community structure observed during early days of the fermentation, as populations responded to the nutritional substrates provided by the *I*. *spicata* plant material. Being a perennial legume, *I*. *spicata* material is relatively high in protein (21.25%) and fibre (50.6%), and therefore provision of these substrates encouraged the proliferation of both Firmicutes and Bacteroidetes, including those often considered to be dominant or core rumen bacteria, classified within the Clostridiales (*Butyrivibrio*, Lachnospiraceae and *Ruminococcus*), Prevotellaceae (*Prevotella*) and the Rikenellaceae RC9 gut group [35,37]. All of these microbes contribute to feed breakdown in the rumen, being metabolically diverse and capable of utilising various different ruminant diets [38,39]. The largely uncultivated Rikenellaceae RC9 gut group has also been identified as being highly abundant in the faecal microbiome of ruminants [40,41]. Our study confirmed that these core rumen bacteria could also utilise the highly fibrous yet also proteinaceous pasture plant, *Indigofera*.

An in vitro fermentation system, however, will never entirely replicate the physiological conditions provided by a living ruminant [42] and not all rumen microbial populations can be introduced into culture [43,44,45]. The fermentation apparatus used in this study could sufficiently simulate rumen-like physiological conditions to successfully cultivate many rumen microbes, through controlling the pH, temperature and maintaining the very low oxygen conditions. Following inoculation of the fermenter system with rumen microbes, an initial decline in microbial diversity was observed and approximately one third of prokaryotes (bacteria and archaea) identified in the original rumen fluid were lost from the system. Despite not being examined in this study, it is also likely that eukaryote populations usually found in pasture-fed ruminants (protozoa and anaerobic fungi) would not have survived the transition to the fermentation system. Despite these technical limitations, a high level of microbial diversity was sustained, as indicated using three diversity measures, and modelling showed an actual increase in diversity during the latter days of the fermentation. It is also likely that by cultivating a mixed microbial population, a rumen-like community structure was maintained, with bacterial species which may rely on metabolites provided by other microbes to grow, proliferating in the conditions provided by the fermentation, for example, the genus *Pyramidobacter*.

Although the microbial populations cultivated in the fermenter were able to maintain the functional capacity to degrade indospicine, even when removed from the environmental conditions provided by the fermenter, the specific bacteria contributing to the breakdown of indospicine could not be determined. Further experimental work in order to isolate and determine the capacity of individual bacterial species to breakdown indospicine and produce the breakdown products 2-APAA and 2-APA, using methodology such as transcriptomics and proteomics, would also be required to verify which enzymatic pathways can be employed by bacteria for the metabolism of this arginine analogue.

The majority of microbial proteolysis in the rumen involves the breakdown of plant proteins and polypeptides, rather than the metabolism of individual (free) amino acids (reviewed by [46,47,48]). Some species in the rumen, which are secondary fermenters and tend to grow on the substrates produced by other rumen microbes, such as *Synergistes jonesii*, have been shown to metabolise free amino acids such as arginine [49]. The amidino functionality present in indospicine is very unusual and no references could be found for microbial degradation of this specific group. As indospicine is an arginine analogue, it can be speculated that the organisms which can catabolise arginine may also be able to breakdown indospicine. Enzymes belonging to the arginine deiminase pathway, including arginine deiminase, ornithine transcarbamylase and carbamate kinase may be involved [50]. This pathway allows the metabolism of arginine to CO_2_ acetate, butyrate, citrulline and ornithine. Similarly, *Treponema* species have reported arginine deiminase activity to produce citrulline, and have potential for utilising indospicine for similar deiminisation to 2-APAA (https://www.uniprot.org/uniprot/Q73QJ2; accessed on 1 March 2021). Literature also suggests that Clostridiales and Bacteroidetes are potential candidates for free amino acid breakdown to produce butyrate and propionate (reviewed by [51]). Notably, in the current study, populations of Clostridiales were shown to significantly increase in relative abundance during the later days of the *Indigofera* fermentation. Indospicine, however, lacks the internal C-N bond present in arginine and even if an organism has an arginine deiminase, this enzyme may not necessarily accept indospicine as a replacement substrate for arginine. It has also been previously demonstrated that indospicine can act as a competitive inhibitor of arginase activity in liver homogenates, and can inhibit the feedback sensitive enzyme of arginine biosynthesis (N-acetylglutamate 5-phosphotransferase) in *Pseudomonas aeruginosa* [52,53]. Thus, the primary fermentation of *Indigofera* plant material and subsequent breakdown of indospicine to 2-APAA and then to 2-APA and any further metabolites may require a consortium of microbial species, with breakdown activity being provided by multiple enzymatic pathways.

This study showed that the fermentation methodology employed was successful in creating a mixed microbial population, adapted to utilising *I*. *spicata* plant material and capable of rapidly degrading the toxic arginine analogue, indospicine. This implies that if harvested and stored correctly, the mixed, rumen-derived microbial populations produced using this fermenter system, could provide the basis of a live microbial treatment or drench, for reducing both the toxic side-effects of *Indigofera* ingestion and the accumulation of indospicine in the meat of ruminant livestock. This would reduce the possibility of ingesting meat contaminated with indospicine and improve the health and welfare of domesticated ruminants grazing extensive pastures containing *Indigofera*.

## 4. Materials and Methods

### 4.1. Nutritional Analysis of Indigofera spicata Plant Material and Preparation of Indigofera Extract

Mature, flowering *Indigofera spicata* plant material was collected from Coopers Plains, Brisbane (Queensland herbarium AQ0797866, Toowong, QLD, Australia), air-dried and milled (1 mm screen) as a single, composite sample including leaves, stems, pods and flowers. A nutritional analysis of this milled plant material was undertaken at Symbio laboratories using National Association of Testing Authorities, Australia (NATA) approved nutritional testing procedures (Table 1).

This plant material was then extracted in order to obtain a highly concentrated solution of indospicine. Briefly, 50 g of freeze-dried and finely ground *Indigofera* plant material was extracted three times with 250 mL MilliQ water. After centrifugation, the supernatant was combined and freeze-dried. The freeze-dried extract was dissolved in sterile, anaerobic water and the indospicine concentration determined before usage in the indospicine degradation assay.

### 4.2. Ethics Approval

All work involving the use of live cattle and the collection of rumen fluid complied with all relevant local animal welfare laws, guidelines and policies, and was conducted at the Queensland Animal Science Precinct at The University of Queensland, Gatton Campus in accordance with the UQ Animal Ethics Approval SAFS/296/17.

### 4.3. Fermentation Experiment

A fermentation was conducted in a Labfors 3 benchtop fermentation system (Infors HT, Bottmingen, Switzerland) using a fermentation volume of three litres. The fermenter vessel was maintained at pH 6.7, 39 °C and continuously bubbled with a mixture of CO_2_:H_2_ (95:5 *v*/*v*) at 1.2 L/min to ensure anaerobic conditions. The total fermentation time was 14 days and commenced using 3 L of a fermenter starter medium containing per L, 165 mL clarified bovine rumen fluid, 0.5 g peptone, 0.5 g yeast extract, 5.0 g NaHCO_3_, 0.5 g glucose, 0.5 g cellobiose, 165 mL mineral solution A, 165 mL mineral solution B, 10 mL volatile fatty acid solution (VFA1), 1.0 mL resazurin and 0.22 g cysteine HCl. The methods for preparation of anaerobic medium and the formulation of mineral solutions A and B, resazurin solution and the VFA1 solution are previously described [54,55]. At the commencement of the fermentation, 14 g of freeze-dried and finely ground *I. spicata* plant material, equivalent to approximately 45 g fresh *I*. *spicata* plant material, was added as substrate for the fermentation. Approximately 1 h after setup, an aliquot (~20 mL) was drawn off to provide a 0 h timepoint which was treated as described below for subsequent timepoints. The initial microbial population of the fermentation was then provided by a 100 mL volume of cryopreserved rumen fluid (25% glycerol, 25% RF medium [56] and 50% rumen fluid sample), collected from a rumen-fistulated Brahman cross steer maintained on an improved pasture diet with hay supplementation at the University of Queensland Gatton Campus.

Thereafter, on each day of the fermentation, half of the fermenter liquid volume (1.5 L) was removed and replaced with 1.5 L of an anaerobic salt solution containing minimal nutrients (fermenter salts solution [57]) and 10 g of freeze-dried and finely ground *I*. *spicata* plant material was added. In addition, on every successive day of the fermentation, four 1 mL volumes of fermentation fluid were transferred into 1.5 mL microcentrifuge tubes and centrifuged at 16,100× *g* for 10 min (Heraeus Pico17 Centrifuge, ThermoScientific, Waltham, MA, USA). The resulting supernatant was then removed into a clean microfuge tube and stored at −20 °C for subsequent UPLC–MS/MS analysis of indospicine, 2-APA and 2-APAA. The remaining cell pellet was stored at −20 °C prior to genomic DNA extraction.

### 4.4. Indospicine Degradation Assay

An indospicine degradation assay was used to verify if microbial degradation of the toxin was occurring throughout the fermentation. At days 5, 9 and 14 of the fermentation, 6 × 10 mL aliquots of fermenter fluid were placed into sterile Hungate tubes [54] and gassed with CO_2_/H_2_ mix prior to sealing in order to provide anaerobic growth conditions. For three of these Hungate tubes, 200 µL of *Indigofera* extract was added to each tube and to the remaining three tubes, 200 µL salts solution was added to each tube. Immediately after setup, 1.0 mL volumes were removed and stored at −20 °C to provide a 0 h timepoint. The Hungate tubes were then incubated at 39 °C with rocking and 1.0 mL samples taken at 0, 9, 24 and 48 h of incubation. These 1 mL volumes were centrifuged at 16,100× *g* for 10 min (Heraeus Pico17 Centrifuge, ThermoScientific, Waltham, MA, USA) and the supernatant transferred into a clean microfuge tube and stored at −20 °C for subsequent UPLC–MS/MS analysis of indospicine, 2-APA and 2-APAA concentrations.

### 4.5. Chemical Analysis of Indospicine and Related Metabolites in Plant Material, Fermenter and Degradation Assay Samples

Indospicine and 2-APAA (>99% pure), both external standards, as well as the internal standard D3-L-indospicine (>99% pure), were synthesized and provided by Prof. James De Voss and Dr. Robert Lang [15,58], The University of Queensland. 2-APA (>99% pure), and heptafluorobutyric acid (HFBA), at ion chromatography grade, were purchased from Sigma Aldrich (Castle Hill, NSW, Australia).

Briefly, samples collected from the fermenter and subsamples, obtained from the indospicine degradation assay, were thawed and diluted 50 times using 0.1% HFBA. A volume of 1 mL of the diluted sample was spiked with 100 µL of the internal standard and filtered through a 0.2 µm syringe filter (Pall, Cheltenham, VIC, Australia).

UPLC–MS/MS was used as previously described [16,17] for the analysis of indospicine, 2-APA and 2-APAA concentrations in samples collected from the fermenter and for subsamples obtained from replicate tubes of fermenter fluid employed in the in vitro indospicine degradation assays.

### 4.6. DNA Extractions and Amplicon Generation from Fermenter Samples

The gDNA from a 1.0 mL frozen rumen pellet sample from each of the fermentation days was extracted using the RBB + C method [59]. The quantity and quality of the extracted gDNA were determined prior to sequencing using the Invitrogen Qubit^®^ (Thermo Fisher Scientific, Waltham, MA, USA) with the dsDNA BR assay kit (Thermo Fisher Scientific, Waltham, MA, USA) as per the manufacturer’s instructions. The extracted gDNA quality was confirmed by 1% agarose gel electrophoresis in TBE buffer and the DNA was visualised using GelRed^®^ stain (Biotium, Fremont, CA, USA). The gDNA samples were diluted to within the range of 10–50 ng/µL in a final volume of 50 µL. The gDNA samples were sent to AGRF for microbial diversity profile sequencing using 16S rRNA gene barcoded amplicons of the 16S rRNA gene V3–V4 region using the forward primer 341F (5′-CCTAYGGGRBGCASCAG-3′) and reverse primer 806R (5′-GGACTACNNGGGTATCTAAT-3′) with overhang adapters and sequenced using the Illumina MiSeq platform to obtain 300 bp paired end reads. The sequencing was undertaken in a single submission of duplicate DNA samples extracted from fermenter fluid aliquots collected on every day of the fermentation, together with a negative control (sample blank) and duplicate subsamples of the cryopreserved rumen fluid used to first inoculate the fermentation.

### 4.7. Amplicon Sequence Analysis and Statistics

The quality of the raw sequence data was initially checked using FASTQC software (https://www.bioinformatics.babraham.ac.uk/projects/fastqc/; accessed on 1 February 2021) then quality filtered, trimmed of barcodes and primers, and size filtered to retain sequences with a minimum length of 200 nt using Trimmomatic version 0.36 [60]. These sequences were then analysed using the Quantitative Insights Into Microbial Ecology 2 (QIIME2) software pipeline package Version 2019.1 [61,62]. The forward and reverse sequence reads were formatted for import into QIIME2 and using the DADA2 software for modelling and correcting Illumina-sequenced amplicon errors [63]. The input sequences were further quality filtered, the forward and reverse reads merged, unique sequences (sequence variants) grouped, and chimeras removed. Negative sequencing controls were also removed from further analysis. For the thirty samples remaining, a total of 681,667 quality filtered, joined reads were used in the downstream sequence analysis with a median of 23,349 sequences per sample. A Feature table containing the counts (frequencies) of each unique sequence in each sample in the dataset (Feature), a representative sequences file and a FeatureData file which maps Feature identifiers in the Feature table to the sequences they represent, was then created. The Feature table was further filtered to remove Features representing <5 sequences, with 1897 Features remaining. A multiple sequence alignment using the Multiple Alignment using Fast Fourier Transform software [64] and a phylogenetic tree was created to relate Features to one another and assign phylogenetic groups to the Feature table. Taxonomy was then assigned using a pre-trained Naïve Bayes classifier trained on the SILVA database (2017 update 132, [65]).

Alpha diversity measures (describing the microbial diversity within a sample) and beta diversity measures (differences in diversity between samples) were calculated using QIIME2 software. Alpha diversity analysis was determined on the basis of three measures: (1) counts of observed species (observed species); (2) Faith phylogenetic diversity (Faith PD); and (3) Shannon entropy of counts (Shannon). For determination of the differences in the microbial communities occurring between samples (beta diversity), the respective metadata files, as well as the table, representative sequence (rep set), and unrooted phylogenetic tree (.tre) files generated using QIIME 2, were imported into the R packages, Phyloseq version 1.30.0 [66] and MixOmics version 6.10.6 [67].

Core microbial communities were determined following taxonomic classification of Features identified using QIIME 2. Features which were present in 100% of samples were designated as “core” microbial communities. For comparison of core microbial communities, Venn diagrams (https://bioinfogp.cnb.csic.es/tools/venny/; Oliveros, J.C., 2007–2015 accessed on 1 March 2021) and lists of microbial populations which were designated as either shared or unique were compiled.

### 4.8. Statistics

The three within-sample microbial diversity measures (observed species, Shannon and Faith phylogenetic diversity), calculated for two replicate samples from each day of the fermentation, were analysed with GenStat v19 (VSN International, 2018). Regression analysis with each alpha diversity measure as the response variate and fermentation day as the explanatory variate was undertaken. A line plus exponential regression was found to fit the data better than either a linear or exponential regression. This was determined using the R^2^ and visual assessment of the fit of the regression.

Using the MixOmics R package [67], an unsupervised analysis with principal component analysis (PCA) [68] (http://mixomics.org/methods/pca/, accessed on 1 March 2021) was conducted using the Feature table data (overall microbial community dataset) generated by QIIME 2, transformed using the centred log ratio (CLR). From the PCA, three components were retained, with the first (principal) component explaining as much of the variability in the microbial community data as possible. The following two principal components (components 2 and 3) explained the remaining variability observed.

For the identification of indicator species and determination of microbial signatures, a Sparse Partial Least Squares Discriminant Analysis (SPLSDA) was undertaken and a supervised analysis and selection of discriminative OTUs was undertaken with a multivariate analysis SPLSDA on three components [69,70,71]. In this way, the most discriminative Features or OTUs (Features being referred to as OTUs within the MixOmics package), that best characterised the days of fermentation were determined. To simplify the effect of fermentation time and increase replication, fermentation days were grouped into four time periods (days 1 to 4, 5 to 7, 8 to 10 and 11 to 14). The outputs of this analysis were also visualized using contribution plots, which listed the most discriminative OTUs, generated based on the coefficient derived from the component analysis. The importance of the respective OTUs in determining the microbial signature was indicated, with the sign on the x axis of each plot representing the positive or negative correlations occurring between the OTUs, relative to the proportions of the others.

## Figures and Tables

**Table 1:** Nutritional analysis of *I. spicata* plant material.

Nutritional Analysis	Concentration (g/100 g or % *w*/*w*)
Dry Matter	92.4%
Ash	14.50
Crude Fibre	23.60%
Dietary Fibre (total)	50.60
Fat	4.70
Nitrogen	3.4%
Protein (N % × 6.25)	21.25%
Sodium	0.025
Phosphorus	0.27
Calcium	2.40
Potassium	1.99
Sulphur	0.308

**Table 2:** Top 20 core populations of highly abundant bacteria identified in fermenter fluid samples collected from two time periods (days 8 to 10; and days 11 to 14) when microbial populations were found to be most stable. If taxonomy could not be assigned to the genus level, the highest designated level of taxonomic classification is shown.

Fermentation Days 8 to 10		Fermentation Days 11 to 14	
Highly abundant core bacteria *	Relative abundance (%) **	Highly abundant core bacteria	Relative abundance (%)
Rikenellaceae RC9 gut group	20.84	Rikenellaceae RC9 gut group	15.65
*Prevotella* 1	11.44	Bacteroidales F082 (uncultured rumen bacterium)	12.39
Bacteroidales F082 (uncultured rumen bacterium)	10.80	*Prevotella* 1	10.90
*Ruminococcus* 1	5.68	*Butyrivibrio* 2	6.51
Prevotellaceae Ga6A1 group	4.75	*Ruminococcus* 1	5.70
Lachnospiraceae	4.36	*Lachnoclostridium*	3.76
*Butyrivibrio* 2	4.27	Lachnospiraceae	3.07
*Lachnoclostridium*	3.14	Clostridiales	2.66
Clostridiales	2.99	Prevotellaceae Ga6A1 group	2.17
*Desulfovibrio*	2.26	*Eubacterium coprostanoligenes* group	2.17
*Anaeroplasma*	2.15	Christensenellaceae R-7 group	1.89
Ruminococcaceae NK4A214 group	1.82	*Anaeroplasma*	1.87
*Fibrobacter*	1.60	Bacteroidales RF16 group	1.85
Lachnospiraceae	1.51	Ruminococcaceae NK4A214 group	1.81
*Eubacterium coprostanoligenes* group	1.19	*Treponema* 2	1.76
*Campylobacter*	1.10	*Campylobacter*	1.58
Christensenellaceae R-7 group	1.10	Prevotellaceae UCG-003	1.50
Lachnospiraceae UCG-009	0.99	*Eubacterium ruminantium* group	1.45
*Eubacterium ruminantium* group	0.91	Ruminococcaceae UCG-014	1.14
Bacteroidales RF16 group	0.87	*Desulfovibrio*	1.09

* Core microbial populations designated as those found in 100% of samples within the respective sample grouping. ** Relative abundance calculated on the basis of the total sequence number for each sample group (105,902 sequences for days 8 to 10; 190,865 sequences for days 11 to 14).

## Data Availability

The metagenomic amplicon sequence datasets generated and analysed in the current study were deposited in the NCBI Sequence Read Archive database under BioProject Accession number PRJNA678115.

## Supplementary Materials

The following are available online at https://www.mdpi.com/article/10.3390/toxins13060389/s1, Tables S1 to S3.
